# Antifungal Testing and High-Throughput Screening of Compound Library against *Geomyces destructans*, the Etiologic Agent of Geomycosis (WNS) in Bats

**DOI:** 10.1371/journal.pone.0017032

**Published:** 2011-03-02

**Authors:** Sudha Chaturvedi, Sunanda S. Rajkumar, Xiaojiang Li, Gregory J. Hurteau, Michael Shtutman, Vishnu Chaturvedi

**Affiliations:** 1 Mycology Laboratory, Wadsworth Center, New York State Department of Health, Albany, New York, United States of America; 2 Department of Biomedical Sciences, School of Public Health, University at Albany, Albany, New York, United States of America; 3 Ordway Research Institute, Albany, New York, United States of America; Institut de Pharmacologie et de Biologie Structurale, France

## Abstract

Bats in the northeastern U.S. are affected by geomycosis caused by the fungus *Geomyces destructans* (*Gd*). This infection is commonly referred to as White Nose Syndrome (WNS). Over a million hibernating bats have died since the fungus was first discovered in 2006 in a cave near Albany, New York. A population viability analysis conducted on little brown bats (*Myotis lucifugus*), one of six bat species infected with *Gd*, suggests regional extinction of this species within 20 years. The fungus *Gd* is a psychrophile (“cold loving”), but nothing is known about how it thrives at low temperatures and what pathogenic attributes allow it to infect bats. This study aimed to determine if currently available antifungal drugs and biocides are effective against *Gd*. We tested five *Gd* strains for their susceptibility to antifungal drugs and high-throughput screened (HTS) one representative strain with SpectrumPlus compound library containing 1,920 compounds. The results indicated that *Gd* is susceptible to a number of antifungal drugs at concentrations similar to the susceptibility range of human pathogenic fungi. Strains of *Gd* were susceptible to amphotericin B, fluconazole, itraconazole, ketoconazole and voriconazole. In contrast, very high MICs (minimum inhibitory concentrations) of flucytosine and echinocandins were needed for growth inhibition, which were suggestive of fungal resistance to these drugs. Of the1,920 compounds in the library, a few caused 50% - to greater than 90% inhibition of *Gd* growth. A number of azole antifungals, a fungicide, and some biocides caused prominent growth inhibition. Our results could provide a theoretical basis for future strategies aimed at the rehabilitation of most affected bat species and for decontamination of *Gd* in the cave environment.

## Introduction

*Geomyces destructans* (*Gd*) causes geomycosis, a devastating disease of hibernating bats that has resulted in the death of tens of thousands if not more than a million individuals of six bat species in the northeastern U.S. The fungus is the only etiologic agent thus far identified that is associated with White Nose Syndrome (WNS) [1], [2]. To date, *Gd* has most seriously affected an insectivorous species, *Myotis lucifugus* (“little brown bat”), but recent studies indicate that it causes high mortality in other bat species and in geographical areas beyond the northeastern U.S. [3]. The projected regional extinction of *M. lucifugus* could lead to a serious imbalance in the ecosystem with unforeseen consequences [3]. There is an urgent need to understand the pathogenesis of geomycosis and to devise control measures to halt and reverse this potential mass extinction.

Any scheme for the treatment of infected bats with antifungal drugs faces a number of challenges. At the outset, there is no information about the effectiveness of the currently available drugs for a fungus that grows at very low temperatures; these drugs are usually effective at the physiological range. An equally challenging task will be the delivery of drugs to an infected population in the wild. Recent studies on the precipitous decline in amphibians due to infection by the chytrid fungus *Batrachochytrium dendrobatidis* have revealed some important insights including the need for the judicious selection of a therapeutic agent [4]. Treatment attempts directed at tadpoles have shown that itraconazole was effective against the fungus, but it caused severe damage to the host [5]. Conversely, the same treatment worked well in a majority of anuran species [6]. Other recent studies revealed that the treatment of fungal infections in amphibians would be best targeted by selecting drugs that function first in the laboratory [7], [8].

The natural habitat of *Gd* is unknown although caves are obviously high on the list because it is believed that hibernating bats become infected in caves that contain *Gd*. Caves harbor unique fungal communities, which are greatly affected by human activity [9], [10]. It is not known if human activities played a role in the initial outbreak and subsequent spread of geomycosis. However, the United States Fish and Wildlife Service has temporarily blocked public access to many caves and mines as a preventative measure aimed at stopping further spread of the disease by humans (http://www.fws.gov/whitenosesyndrome/pdf/NWRS_WNS_Guidance_Final1.pdf). Many of the affected caves are important for tourism and recreational activities. Thus, it might become necessary to decontaminate heavily infested caves and other natural sites using safe and effective chemicals that selectively eradicate *Gd*. This study aimed to identify antifungal drugs and antiseptic compounds for potential treatment of geomycosis and decontamination of *Gd*.

## Results

**Figure 1:**
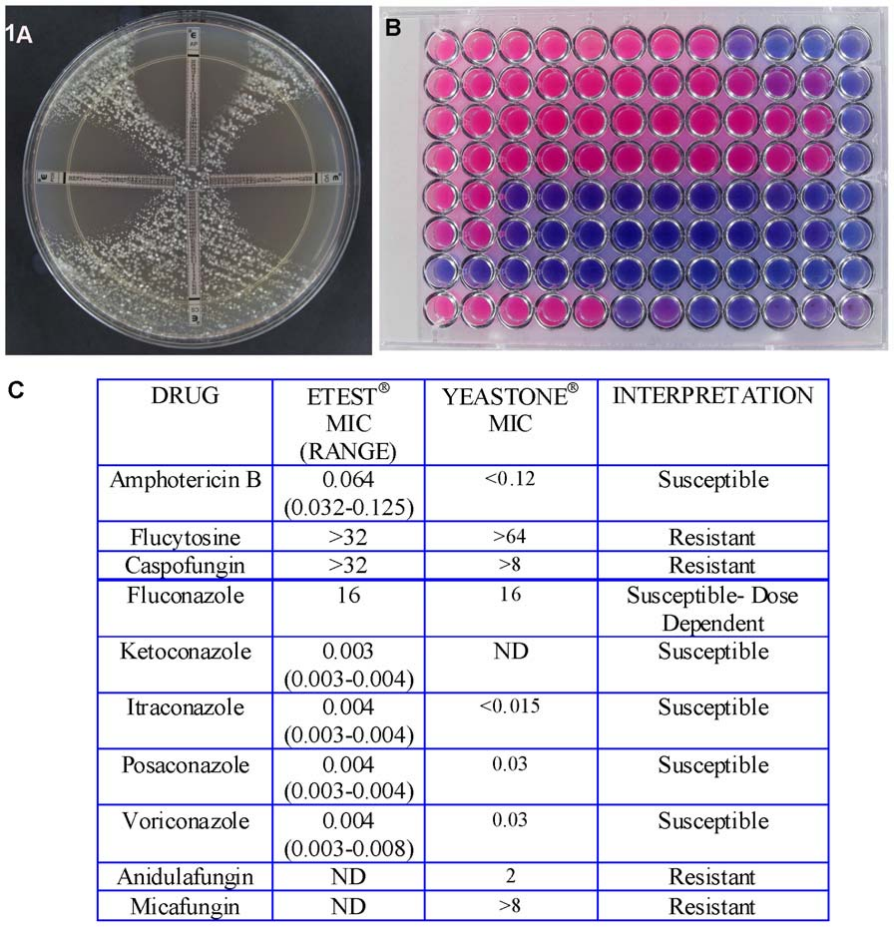
Antifungal susceptibility testing of *Geomyces destructans*. Panel A shows an Etest plate incubated for 10 days at 15°C with the following patterns clockwise from top: amphotericin B (low MIC suggestive of susceptibility) , voriconazole (low MIC suggestive of susceptibility), caspofungin (high MIC suggestive of resistance), and posaconazole (low MIC suggestive of susceptibility). Panel B includes a representative YeastOne® plate with magenta microwells showing fungal growth and blue microwells showing growth inhibition. Panel C includes a summary of results obtained from two devices with susceptibility to amphotericin B, fluconazole, ketoconazole, itraconazole, posaconazole and voriconazole. Suggestive resistance to flucytosine and caspofungin was found in testing using E-test and YeastOne®, and suggestive resistance to anidulafungin and micafungin was found in testing using YeastOne®.

All five strains of *Gd* had similar antifungal susceptibility profiles in our tests irrespective of the test devices used (Figure 1A–1C). Amphotericin B and four azoles (itraconazole, ketoconazole, posaconazole and voriconazole) were effective at low MICs against *Gd* strains while fluconazole MICs were suggestive of Susceptible-Dose Dependent (S-DD) pattern. Flucytosine and all three echinocandins (caspofungin, anidulafungin and micafungin) exhibited relatively high MICs suggestive of resistance (Figure 1C). Unlike pathogenic fungi, susceptibility testing of *Gd* strains required prolonged incubations for 10–21 days at 15°C and 6°C, respectively. The antifungal susceptibility testing proceeded relatively faster at 15°C than at 6°C as expected due to the better growth of the fungus seen at 15°C [2]. It is noteworthy that YeastOne® microdilution test yielded results on an average of 14 days while Etest was positive in 10 days. This difference in turnaround time was accentuated when the tests were done at 6°C (Figure S1).

**Figure 2:**
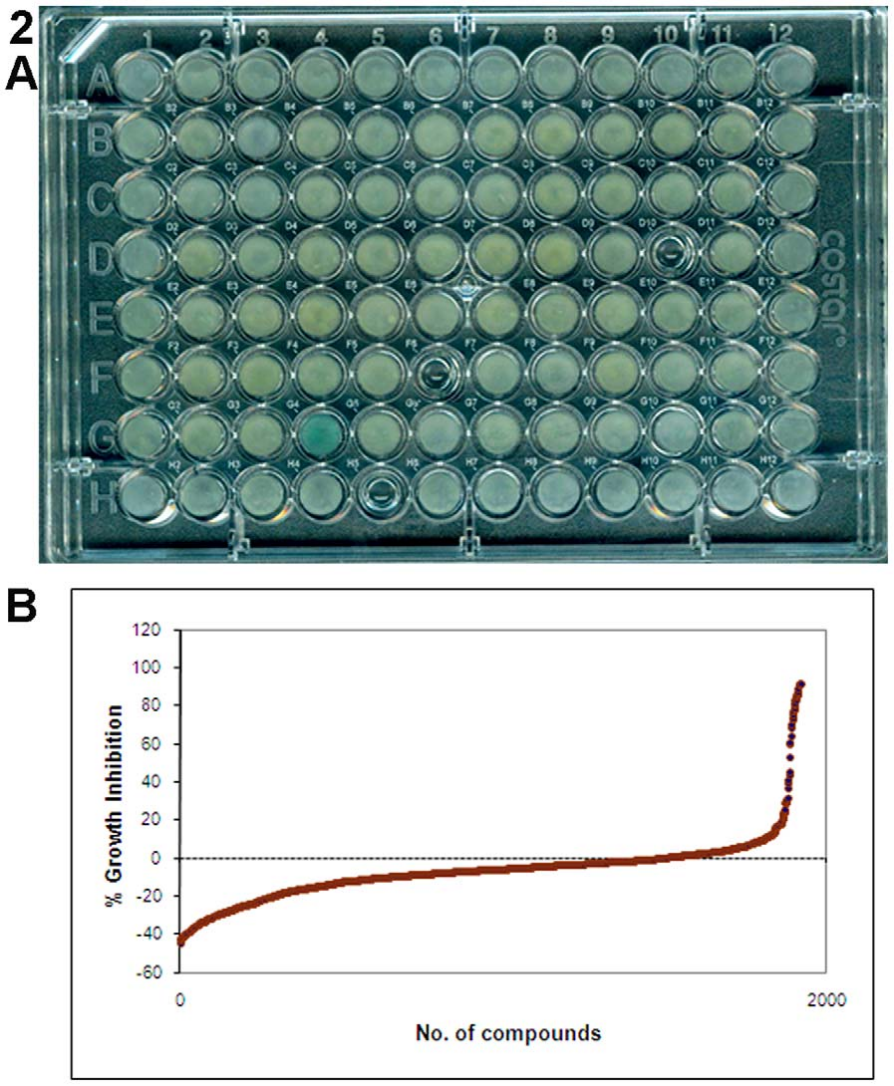
High Throughput Screening (HTS) of the compound library for *G. destructans* growth inhibition. Panel A illustrates a 96-well microplate used in HTS with growth inhibitions seen at position D10 (benzalkonium chloride-86%), F6 (chloroacetoxyquinoline-87%) and H5 (methylbenzethonium chloride- 91%) compared to the growth controls in columns 1 and 12. Panel B provides an overview of the entire screen of 1,920 compounds with a majority of compounds having no inhibitory effect on the growth of *G. destructans*.

**Figure 3:**
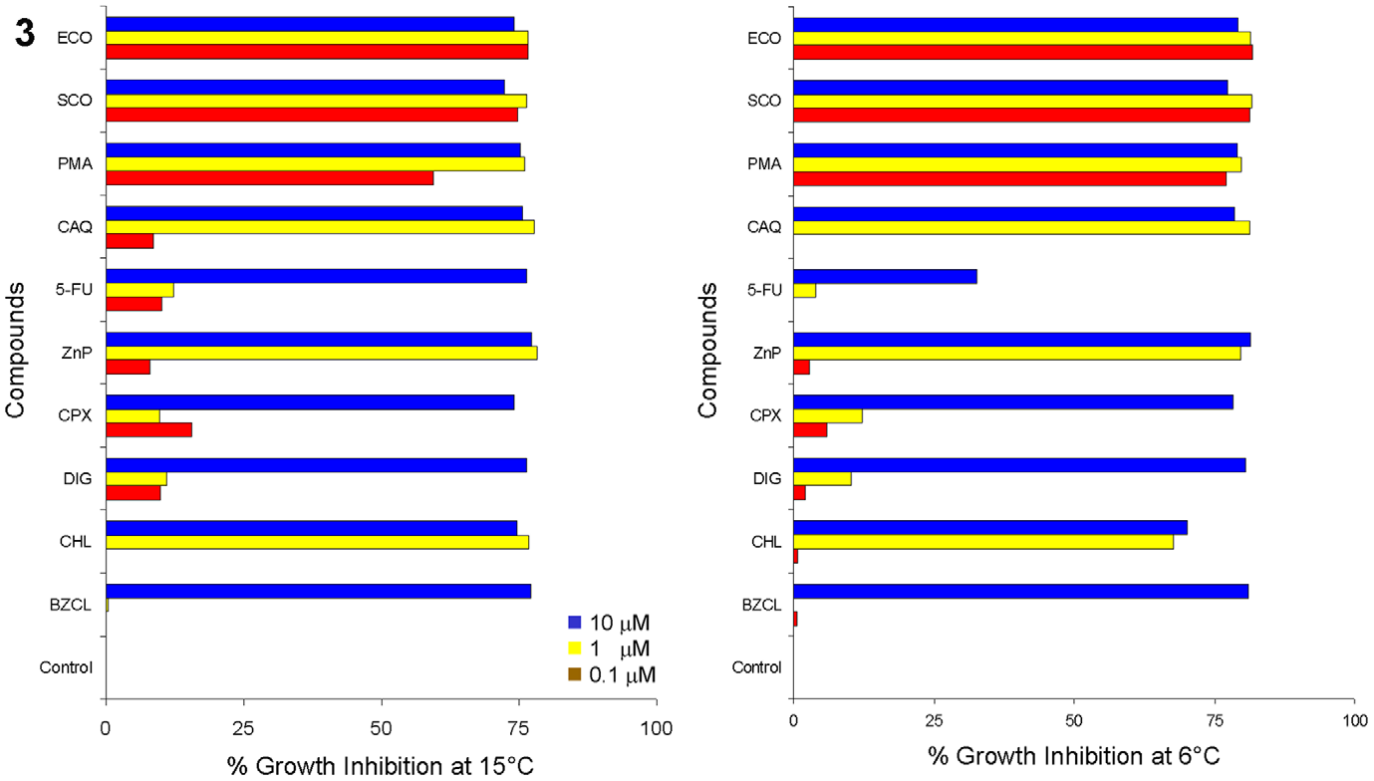
Summary of secondary screening for *G. destructans* growth inhibition. The results shown were obtained at 15°C and 6°C with 10 promising compounds tested ten-fold and hundred-fold lower concentrations than used in the initial screen. Note that two topical azole antifungals and a fungicide were most effective at all concentrations tested. (Abbreviation: ECO econazole nitrate, SCO sulconazole nitrate, PMA phenylmercuric acetate, CAQ chloroacetoxyquinoline, 5-FU fluorouracil, ZnP pyrithione zinc, CPX ciclopirox olamine, DIG digitonin, CHL chloroxine, BZCL benzalkonium chloride).

The results of HTS identified 27 compounds (1.4% of 1,920 tested), which caused 50% to- greater than 90% inhibition of *Gd* growth at 15°C while other compounds tested had no inhibitory effect (Figure 2A, 2B and Table S1). Of the 27 compounds, ten were randomly selected for further analysis using 10 µM original concentration and ten-fold (1.0 µM) and hundred-fold (0.1 µM) lower concentrations in the microtiter plates. These compounds and *Gd* were incubated both at 15°C and 6°C. This secondary screen confirmed excellent growth inhibitory profiles of selected compounds at both temperatures (Figure 3 and Tables 1, S2, S3). Not surprisingly, antifungals (econazole, sulconazole, chloroacetoxyquinoline, pyrithione zinc, ciclopirox olamine, and chloroxine), fungicide (phenylmercuric acetate), and biocide (benzalkonium chloride) caused prominent growth inhibition. Notably, pyrimidine analog (fluorouracil) and a biomembrane disruptor (digitonin) were also highly effective.

**Table 1:** List of compounds from SpectrumPlus Compound Library found highly effective at growth inhibition of *Geomyces destructans* at 6°C and 15°C.

#	Compounds	Abbreviation	Chemical Description	Mode of action
1	Econazole nitrate	ECO	Imidazole class	Inhibition of 14 <alpha>-sterol demethylase and the subsequent blockage of ergosterol biosynthesis; antifungal drug for Athlete's foot, ringworm, etc.
2	Sulconazole nitrate	SCO	Imidazole class	Inhibition of 14 <alpha>-sterol demethylase and the subsequent blockage of ergosterol biosynthesis; antifungal drug for treatment of Athlete's foot, ringworm, etc.
3	Phenylmercuric acetate	PMA	Organomercury compound	Fungicide
4	Chloroacetoxyquinoline	CAQ	Hydroxyquinoline derivative	Antibacterial, antifungal
5	Fluorouracil	5-FU	Pyrimidine analog (heterocyclic aromatic compound)	Noncompetitive inhibition of thymidylate synthase; synthesis of aberrant mRNA and the subsequent inhibition of protein synthesis; cancer drug
6	Pyrithione Zinc	ZnP	Coordination complex of Zinc	Antifungal & antibacterial agent
7	Ciclopirox olamine	CPX	Synthetic compound (6-cyclohexyl-1-hydroxy-4-methylpyridin-2(1H)-one)	Topical antifungal for superficial mycoses
8	Digitonin	DIG	Glycoside (from *Digitalis purpurea*)	Detergent, Biomembrane disruptor
9	Chloroxine	CHL	Halogenated hydroxyquinoline	Antibacterial, Antifungal
10	Benzalkonium chloride	BZCL	Quaternary ammonium group	Antiseptic, biocide, surfactant,

## Discussion

A remarkable observation in this study is that the antifungal drugs are active at the psychrophilic range, a fact not previously documented in the published literature. *Geomyces destructans* was susceptible to amphotericin B and azoles at MICs that are similar to the values reported for pathogenic yeasts and filamentous fungi [11], [12], [13]. Azoles and amphotericin B represent some of the most effective antifungal drugs that act by inhibition of ergosterol biosynthesis or by binding to ergosterol in the plasma membrane. Our results expand their broad-spectrum of activity to a psychrophilic pathogen. Thus, we have identified many antifungal drugs that work well in the laboratory against *Gd* at temperature ranges likely to be encountered in bat hibernacula. This observation could be the basis for future trials of these drugs in efforts aimed at the rehabilitation of most affected bat species.

The observed high flucytosine MIC for *Gd* was suggestive of resistance. Such resistance to flucytosine is fairly widespread among filamentous fungi and the resistance mechanisms are manifold. In studies with *Aspergillus* species, it was found that resistant strains were not defective in the drug uptake nor in its conversion to 5-flourouracil, but they utilized cytosine as a nitrogen source to circumvent the drug effect [14]. Another noteworthy finding of the present study is the high echinocandins MICs for *Gd*; this suggests intrinsic resistance similar to other filamentous pathogens such as *Fusarium* species, *Scedosporium prolificans*, and zygomycetes [13], [15]. This finding suggests that *Gd* shares an important characteristic with fungal pathogens, which cause serious and intractable infections in humans. Echinocandins-resistance in the aforementioned fungi is due to specific mutations in the membrane protein Fks1, which encodes β-1,3-glucan synthase catalytic subunit crucial for cell wall synthesis [16]. It would be interesting to investigate if a similar or a novel mechanism of echinocandins-resistance exists in *Gd*.

The yield of effective compounds in HTS relative to the number of compounds tested is similar to other HTS for antifungal activities [17], [18]. We believe that the psychrophilic conditions needed for this screen and the innate ability of *Gd* to grow in the presence of a diverse group of chemicals limited the outcome to a few well-known fungal inhibitors. The list of the most effective compounds included antifungals, fungicide and biocides. In contrast to these known inhibitors, the high susceptibility of *Gd* to fluorouracil was notable and unexpected since *Gd* was found to be resistant to flucytosine. This observation suggested that the fluorouracil mode of action in *Gd* might include inhibition of RNA and DNA synthesis similar to the dual mode of actions reported for other fungi [14], [19], [20]. By the same logic, it is more likely that the observed flucytosine-resistance in *Gd* involves either defects in the uptake of this drug or its inefficient conversion to 5-fluorouracil (5-FU) and 5-fluorodeoxyuridine monophosphate (5-Fd-UMP).

The effective biocides and fungicides identified in this study are currently in use, and therefore, could potentially be useful for the decontamination of *Gd* infested sites. This is a positive step as earlier publications on decontamination of sites harboring pathogenic fungi such as *Histoplasma capsulatum* used formalin, which is not an option at present due to the associated toxicity [21]. However, any proposal for the decontamination of infested sites requires a careful balance considering the likely deleterious effects on the environment. It is worth repeating that caves support a rich diversity of life including many species of fungi and bacteria [10], [22]. So far, extensive published data is only available for benzalkonium chloride. This chemical was found to be an effective disinfectant for household items contaminated with the dermatophytic pathogen *Microsporum canis* [23]. Benzalkonium chloride has been used for many years in the famous Lascaux cave (Montignac, France) to control *Fusarium solani* infestation. Although effective, this treatment has resulted in the replacement of native bacterial communities with more resistant bacteria [24]. The long-term environmental effects of such biocide-induced changes remain unknown. The effective inhibition of *Gd* growth by phenylmercury acetate suggests that organomercury fungicides could also be effective agents for the decontamination of natural sites. Similar to the concerns with biocides, any future remedial plans involving fungicides would require further refinements to ensure that the non-target fungi and other living forms are not harmed [25], [26]. In conclusion, a number of antifungal drugs and inhibitory compounds were systematically identified in this study; these results could serve as a starting point for the selection of appropriate agents to treat *Gd*-infected bats in rehabilitation centers and to decontaminate heavily infested sites.

## Materials and Methods

### Antifungal susceptibility testing

Two different commercial devices were used for antifungal testing. These devices yield reproducible results and are reported to be good alternatives to standard methods, which might be too cumbersome for many laboratories working with *Gd* [12], [27], [28], [29]. Five strains of *Gd* characterized in an earlier study were used in the susceptibility tests [2]. In brief, *Gd* strains were grown on potato dextrose agar for 10 days at 15°C. Spore suspensions were prepared in sterile water, adjusted to OD_530_ = 0.1. For testing with Etest strips, diluted spore suspension was streaked on to RPMI/MOPS agar and the Etest strips were placed per the manufacturer's instruction (bioMerieux, Inc., Durham, NC). For 96-microwell dilution tests, the YeastOne® with Anidulafungin, Caspofungin and Micafungin panels were used (Part #YO-9, TREK Diagnostic Systems, Inc., Cleveland, OH). The susceptibility patterns of antifungals were assessed 10–14 days post-incubation at 15°C and 21–28 days post-incubation at 6°C. We have previously reported that *Gd* grows faster at 15°C than at 6°C without any apparent changes in its morphology or secretory enzymes [2].

### High-throughput screening (HTS)

The method used for HTS was done as previously described by Li et al. [30]. A SpectrumPlus (MicroSource) library representing 1920 structurally diverse compounds including known drugs, experimental actives, and pure natural products, was used. The compounds were distributed in sterile 96-microwell plates with a Sciclone ALH 3000 workstation (Caliper Life Sciences, Hopkinton, MA). The control well of the 96-microwell plate contained 100 µl of 0.1% DMSO in sterile water+100 µl of *Gd* spore suspension (OD_530_ = 0.1) in 2× yeast nitrogen glucose broth (2% yeast nitrogen base+4% glucose; YNGB). All other wells contained 100 µl of different drugs (10 µM concentration in 0.1% DMSO)+100 µl of spore suspension in 2× YNGB. The growth inhibition was assessed spectrophotometrically after 10 days of incubation at 15°C. Inhibition of the compounds was calculated as percentages from the ratio of absorbance for *Gd* with compound wells compared to no compound in 0.1% DMSO alone. Secondary screening of selected compounds proceeded similar to the primary screen except that the 10- and 100-fold lower concentrations of the effective compounds identified in the initial screens were tested both at 15°C and 6°C.

## Supporting Information

Figure S1 **Antifungal susceptibility testing of** ***Geomyces destructans*** **.** Panel A. Etest plate showing *G. destructans* antifungal susceptibility and resistance at 6°C incubated for 21 days with the following susceptibility patterns, clockwise from top: amphotericin B (susceptible), voriconazole (susceptible), caspofungin (resistant) and posaconazole (susceptible). Panel B. YeastOne® plate with magenta microwells showing *G. destructans* growth and blue microwells with growth inhibition at 6°C. Well A1 is positive control, A2–A11 anidulafungin, B1–B11 micafungin, C1–C11 caspofungin, D1–D11 flucytosine, E1–11 posaconazole, F1–F11 voriconazole, G1–G11 itraconazole, H1–H12 fluconazole and Column 12, Row A–G amphotericin B.
(DOCX)

Table S1 **Results of high throughput screen (HTS) of 1920 compounds in SpectrumPlus Library for growth inhibition of** ***Geomyces destructans*** **.**
(XLSX)

Table S2 **Results of secondary screen of** ***Geomyces destructans*** **against 10 selected compounds at 15°C.**
(XLSX)

Table S3 **Results of secondary screen of** ***Geomyces destructans*** **against 10 selected compounds at 6°C.**
(XLSX)
