# Supplementary figures and images for: Antifungal Testing and High-Throughput Screening of Compound Library against Geomyces destructans, the Etiologic Agent of Geomycosis (WNS) in Bats
Source: PLoS One. 2011 Mar 2;6(3):e17032. doi: 10.1371/journal.pone.0017032 (PMC3047530; doi:10.1371/journal.pone.0017032)

**Fig S1**

A


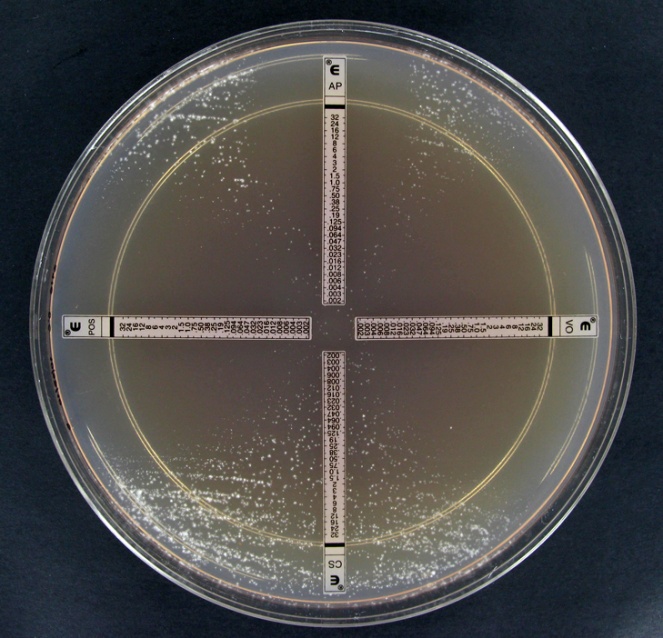


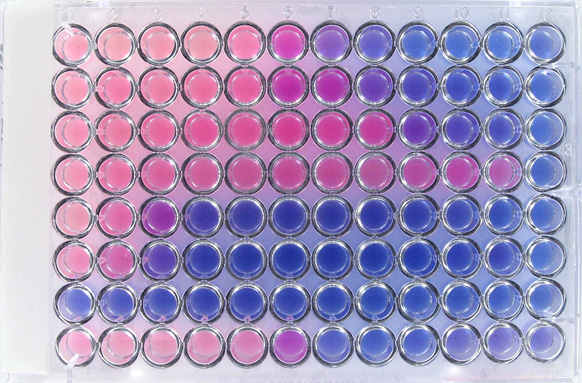


B

Supplement: Figure S1 — Antifungal susceptibility testing of Geomyces destructans . Panel A. Etest plate showing G. destructans antifungal susceptibility and resistance at 6°C incubated for 21 days with the following susceptibility patterns, clockwise from top: amphotericin B (susceptible), voriconazole (susceptible), caspofungin (resistant) and posaconazole (susceptible). Panel B. YeastOne® plate with magenta microwells showing G. destructans growth and blue microwells with growth inhibition at 6°C. Well A1 is positive control, A2–A11 anidulafungin, B1–B11 micafungin, C1–C11 caspofungin, D1–D11 flucytosine, E1–11 posaconazole, F1–F11 voriconazole, G1–G11 itraconazole, H1–H12 fluconazole and Column 12, Row A–G amphotericin B. (DOCX) [file pone.0017032.s001.docx]
